# Supplementary material for: Exploring Information Exchange between Thesium chinense and Its Host Prunella vulgaris through Joint Transcriptomic and Metabolomic Analysis
Source: Plants (Basel). 2024 Mar 12;13(6):804. doi: 10.3390/plants13060804 (PMC10975001; doi:10.3390/plants13060804)
Supplement: Supplementary file 1 [file plants-13-00804-s001.zip › plants-2859357-Supplementary Material Figure.pdf]

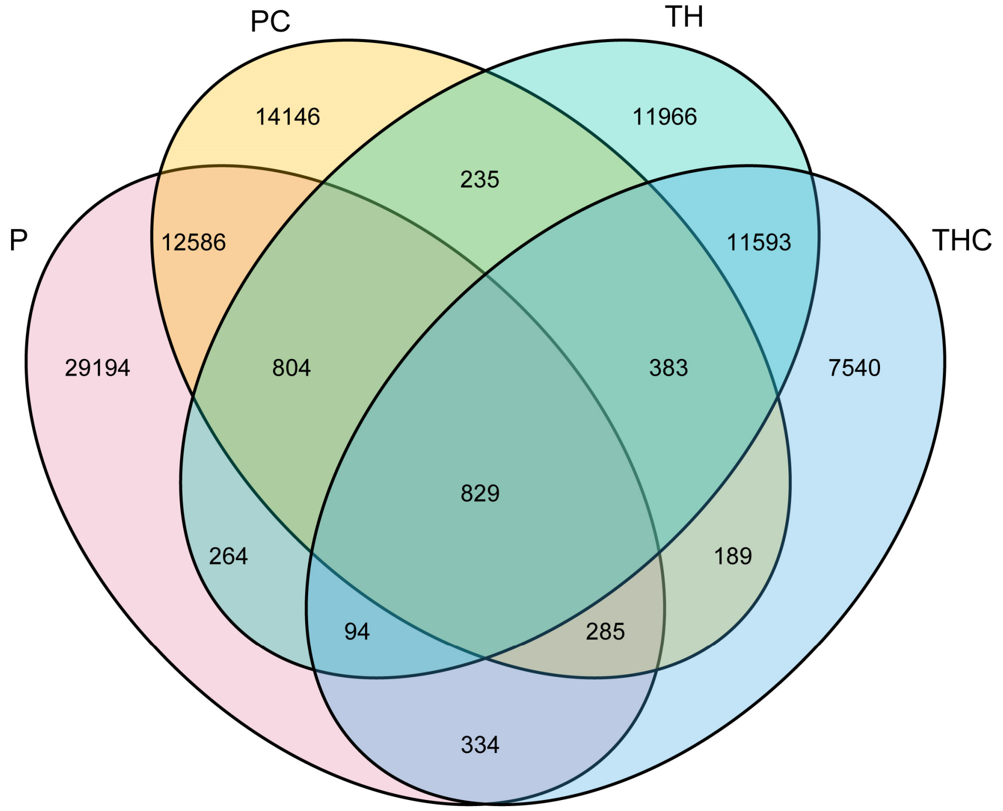

Figure S1. Venn diagrams showing common and unique sets of transcripts in *T. chinense* and its host *P. vulgaris* following parasition.
